# A perspective on life-cycle health technology assessment and real-world evidence for precision oncology in Canada

**DOI:** 10.1038/s41698-022-00316-1

**Published:** 2022-10-25

**Authors:** Dean A. Regier, Samantha Pollard, Melanie McPhail, Tania Bubela, Timothy P. Hanna, Cheryl Ho, Howard J. Lim, Kelvin Chan, Stuart J. Peacock, Deirdre Weymann

**Affiliations:** 1 Canadian Centre for Applied Research in Cancer Control (ARCC), Cancer Control Research, BC Cancer, Vancouver, BC Canada; 2 School of Population and Public Health, Faculty of Medicine, University of British Columbia, Vancouver, BC Canada; 3 Faculty of Health Sciences, Simon Fraser University, Burnaby, BC Canada; 4 Department of Oncology, Queen’s University, Kingston, ON Canada; 5 Department of Public Health Science, Queen’s University, Kingston, ON Canada; 6 Department of Medical Oncology, BC Cancer, Vancouver, BC Canada; 7 Department of Medicine, University of British Columbia, Vancouver, BC Canada; 8 Sunnybrook Health Sciences Centre, Toronto, ON Canada

**Keywords:** Cancer genomics, Health care economics, Outcomes research

## Abstract

Health technology assessment (HTA) can be used to make healthcare systems more equitable and efficient. Advances in precision oncology are challenging conventional thinking about HTA. Precision oncology advances are rapid, involve small patient groups, and are frequently evaluated without a randomized comparison group. In light of these challenges, mechanisms to manage precision oncology uncertainties are critical. We propose a life-cycle HTA framework and outline supporting criteria to manage uncertainties based on real world data collected from learning healthcare systems. If appropriately designed, we argue that life-cycle HTA is the driver of real world evidence generation and furthers our understanding of comparative effectiveness and value. We conclude that life-cycle HTA deliberation processes must be embedded into healthcare systems for an agile response to the constantly changing landscape of precision oncology innovation. We encourage further research outlining the core requirements, infrastructure, and checklists needed to achieve the goal of learning healthcare supporting life-cycle HTA.

## Introduction

Regulatory and reimbursement bodies rely on health technology assessment (HTA) evidence and deliberation to promote equitable and efficient healthcare systems. Advances in precision oncology challenge HTA. Precision oncology advances are rapid, often tumour or disease agnostic, and involve small groups of patients that are clinically evaluated without a contemporary comparison group. Precision oncology has demonstrated correlative health improvements in a few applications^1,2^, but its reach to patients remains limited in routine clinical care^3^, in part due to an insufficiency of evidence and inadequate evaluative processes to support implementation. In this perspective, we describe a life-cycle evaluative approach to precision oncology based on real world data (RWD) collected from a learning healthcare system (LHS)^4^. RWD are data on patient and health system outcomes that are routinely collected from a variety of sources. If the HTA process is appropriately designed and based on local priorities, it will support LHS and provide a platform for generating RWD, which enables real world evidence (RWE). RWE is evidence regarding the benefits, risks, and cost-effectiveness of health technologies based on RWD.

## Managing uncertainty is critical

Evidence generation for precision oncology often relies on non-randomized master protocol trials or randomized controlled trials (RCTs) with limited follow-up or intermediate endpoints^5,6^. Among the many resource use considerations, the cost of comprehensive genomic profiling (CGP) and its associated targeted therapies are substantial^7^. Trials designed to demonstrate clinical activity and safety but not causal efficacy or changes in quality and length of life pose considerable problems for the quantification of effectiveness and cost-effectiveness, where the latter is reliant on incremental costs and quality adjusted life years (QALYs)^8^. The confluence of limited patient numbers, rapidly changing technology cost, non-traditional trial designs, and short-term outcomes data are incongruent with HTA, thus limiting the translation of precision oncology and CGP more generally into routine clinical practice.

An exemplar of the challenges facing precision oncology HTA is Neurotrophic Tyrosine Receptor Kinase (NTRK) inhibitors for solid tumours with an NTRK gene fusion. In Canada, the Canadian Agency for Drugs and Technology in Health (CADTH) did not initially recommend reimbursement for an NTRK inhibitor on the basis of: uncertain magnitude of clinical benefit and cost-effectiveness, the heterogeneity inherent in evaluating tumour agnostic therapies, and a lack of RCT-generated comparative evidence^9^. CADTH’s recommendation subsequently evolved with a conditional positive recommendation with price reduction, noting that if jurisdictions are required to pay for CGP then there is no price for the anticancer drug that would make it cost-effective. In the United Kingdom, the National Institute for Health and Care Excellence (NICE) recommended that this same therapy only be funded through the cancers drug fund with a managed access agreement that gathers additional data to address clinical evidence uncertainties^10^. Common amongst this and other precision oncology exemplars is significant decision uncertainty, where the magnitude and precision of effects and impact on expenditures are largely unknown, making the opportunity cost (or healthcare system economic value) of diverting constrained resources to the intervention impossible to articulate.

Internationally, stakeholders representing HTA bodies recognize that mechanisms for managing uncertainty are critical, particularly in light of significant clinician and patient demand for innovative therapeutics^11^. They have concluded that HTA needs to evolve to reflect the realities of current and future healthcare innovation ecosystems. While health economists have long published methods for managing uncertainty^12^, renewed interest in RWD is advancing conversations about RWE for life-cycle evaluation^13,14^. Life-cycle HTA endeavours to assess the health, economic, and societal impacts of a technology across its life-cycle, from research and development to obsolescence. Such evaluative approaches need to be supported by regulatory and legal frameworks, in addition to processes and methods that aid iterative decision-making. Hereafter we examine the concept of LHS and its role in generating RWE throughout the technology life-cycle. We then propose a life-cycle evaluation framework and discuss relevant future considerations for implementing life-cycle HTA.

## Learning healthcare decisions

Regulatory and reimbursement decisions should be evidence-based. Decision-making processes are deliberative, using an evidentiary package that may include: evidence of safety, clinical effectiveness, cost-effectiveness, patient value, and implementation considerations. Critical deliberations of safety and clinical effectiveness are based on the epidemiological hierarchy of evidence^15^, together with reference case guidelines for health economic evaluation^16,17^. Precision oncology interventions are rarely supported by evidence at the pinnacle of the hierarchy, namely RCTs and meta-analyses of RCTs^6^. Instead, evidence based solely on single-arm studies is used. Single-arm studies cannot establish comparative causality and unbiased effect estimation, leading to uninterpretable evidence. Such uninterpretable evidence should be supplemented to support regulatory or reimbursement deliberations. Additional evidence can be generated through concatenating healthcare systems’ RWD with CGP and trial data.

### Real world data are generated as part of a learning healthcare system

RWD are appropriate for use in regulatory and reimbursement decisions that deliberate on value. Quality RWD generation, however, depends on the design and implementation of LHS. LHS collect and evaluate data and apply evidence to improve patient health; drive discovery as a result of patient-centred care; and ensure innovation, quality, safety, and value in healthcare^18^. In the context of precision oncology, an LHS would longitudinally integrate CGP information with other health data, including: information on patient characteristics, disease characteristics, resource use, treatment, patient-reported outcomes, adverse events, and clinical health outcomes. The use of RWD in an LHS complements, but does not replace, evidence-informed medicine. Both approaches support evidence-driven care and shared decision-making for improved population health^19^.

LHS characteristics have been reported elsewhere^20^; common elements include specifying standards for: fit-for-purpose RWD, technology enabling data curation and architecture, and established governance frameworks, including with respect to ethics, privacy, data security, and law. Decision-grade data generation is premised on health record data systems that enable decisions. These systems are complex, and for most healthcare systems will require improvements in: data architecture and consensus on data use for research and implementation; quality data collection and curation that brings together siloed data sources to inform better decisions, both at the health system reimbursement level and in the regulatory context; and improved computational resources so that data scientists can develop, validate, and deploy approaches, for example, in artificial intelligence and natural language processing.

The use of RWD in decisions has been limited in practice. Healthcare systems face legal, structural, and operational barriers in the development of LHS. For example, generation and use of RWD may be impeded by data stewards’ conservative interpretation and implementation of legislation that governs privacy and health information. Systematic use of RWD has been largely confined to individual clinical decision-making, hospital performance reporting, and quality improvement, all of which work together to enshrine the longstanding problem of a lack of integration of research into LHS^21^. Research is key to LHS, and methods improvements are needed for real world evaluative study designs to enable iterative learning and continual system improvement^21^. Along these lines, we argue that research-enabled RWE together with iterative life-cycle evaluation for HTA is a critical and undeveloped component of LHS.

## Life-cycle assessment

**Fig. 1:**
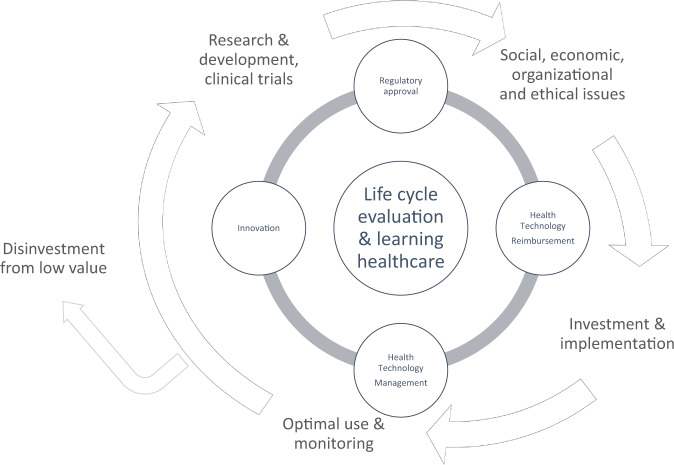
Stages of the technology life-cycle and learning healthcare. Life-cycle stages of drugs include: drug discovery and development (innovation), preclinical and clinical research, regulatory approval based on safety and efficacy, health technology assessment (investment, implementation), health technology management (optimal use), and disinvestment from low-value technologies. Learning healthcare generates and applies evidence to improve patient health; drive discovery as a result of data from patient care; and ensure innovation, quality, safety, and value for money in healthcare.

Life-cycle evaluation can be applied at any stage: from innovation to regulatory decisions, to reimbursement, re-appraisal, and disinvestment (Fig. 1)^22,23^. Each life-cycle phase can draw from complementary HTA methodologies. For example, in the innovation phase (discovery and early phase clinical trials), methods that combine business and economics, including early-stage economic evaluation (what is the range of a cost-effective drug price?) and real-options analysis (should we continue to invest in research?), inform the expected attractiveness of investing in phase II/III clinical trials. Prior to reimbursement, interventions with uncertain clinical trial evidence may be eligible for managed access, which could support conditional access to technologies whose effectiveness and cost-effectiveness are uncertain, providing time-limited access to patients, while generating evidence to decrease decision uncertainty^24–27^. In the post-reimbursement phase, continual surveillance and health technology management provide the opportunity for comparative evaluation using quasi-experimental comparisons of eligible versus ineligible patient cohorts, including through matched and pre/post study designs, such as propensity score or genetic matching^28^ or interrupted time series analysis^29^. The outcomes of these phases can inform disinvestment in expected low-value technologies, freeing resources for other areas of innovation or healthcare. Throughout each life-cycle phase, patients, families, and the public should be engaged as partners and participants in research. Their participation ensures that evidence produced and subsequent reimbursement decision-making meet their needs and preferences.

### A precision oncology life-cycle health technology assessment framework

Generating life-cycle evidence to support timely and affordable access to promising technologies requires careful thought around an evidence and deliberation framework. We turn now to an advanced approach for life-cycle HTA, where there is initial full or conditional regulatory approval for the target technology, combined with uncertainty in comparative effectiveness and cost-effectiveness. We define life-cycle HTA as the standardization of data collected and methods needed to inform life-cycle appraisal, re-appraisal, and de-adoption of health technologies, all conducted within a living and adaptive LHS that periodically examines the value of continued research and evaluation in light of an evolving evidence base. Accordingly, life-cycle HTA is premised on an LHS that is iterative and ongoing, with data that aids serial decision-making. Life-cycle HTA differs from traditional HTA, which focuses on static estimates of effectiveness and expected present value^30^.

**Fig. 2:**
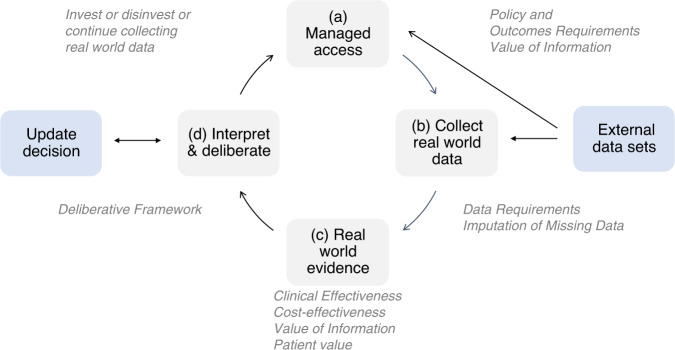
A life-cycle health technology assessment process framework. Life-cycle health technology assessment is the standardization of data collected and methods needed to inform life-cycle appraisal, re-appraisal, and de-adoption of health technologies. The framework includes: managed access for conditional support subject to collecting real world evidence; collecting real world data and concatenating that data with external data; generating real world evidence for decisions, and interpreting the evidence to decide on adoption, disinvesting from managed access, or continuing to collect data.

Our life-cycle HTA process framework is depicted in Fig. 2. The supporting criteria critical to each element of the framework are in Table 1. This framework is directed to generate and evaluate evidence for: (1) technologies with regulatory approval but that are not yet reimbursed, where RCT evidence is either based on small patient numbers or when RCT evidence will not be pursued because of disease rarity or lack of incentivization (e.g., patent expiry); or (2) reimbursed technologies, where there is uncertainty in comparative value, and where technology management is important for sustainability. Framework components are: (a) managed access that defines the time horizon and pricing conditions of real-world healthcare system trialing (including zero-cost or discounted cost drug provision); (b) collecting core data elements for RWD, including leveraging external data; (c) RWE generation to determine comparative effectiveness, cost-effectiveness (net-benefit), and the value of conducting additional research; and (d) interpretation of data and updating of decisions, including investment, continued evaluation, or disinvestment from managed access. Each life-cycle HTA step is briefly examined below in the context of CGP-directed precision oncology.

**Table 1:** Criteria and key considerations for a life-cycle health technology assessment approach to precision oncology.

Framework element	Criteria for life-cycle health technology assessment	Key criteria considerations
Managed access	• Policy and outcome requirements are defined by stakeholders	Inclusion criteria • Technologies have regulatory approval but are not reimbursed and RCT evidence is based on small patient numbers or RCT evidence will not be pursued because of disease rarity or • There is uncertainty in comparative value for reimbursed technologies, and where technology management is important for sustainability. Evaluation: • Conditions of managed access agreement are clearly defined
Collect real world data	• A defined and achievable core data set to support deliberation • Missing data in the core dataset is addressed • External data sets are leveraged to address uncertainty	Healthcare system data criteria • Decision-grade real world data is collected, audited, and mapped to core data requirements • Infrastructure supports data collection or abstraction of reliable data External data • External data is audited for quality and mapped to core data requirements Missing data • A missing data strategy for core data elements is articulated and acceptable to decision-makers
Real world evidence	• Clinical effectiveness • Cost-effectiveness • Patient value • Value of collecting additional data	Study design criteria • Study design is supported by real world data generated from a learning healthcare system • Study design enables causal inference of outcomes • Study design is acceptable to decision makers Uncertainty • Value of information analyses articulate whether there is continued value to collecting additional data given new data
Interpret and deliberate	• A deliberative process examines the evidence and the uncertainty around the evidence • A decision is made to invest, disinvest, or continue managed access	Process criteria • The process is structured to be consistent and transparent • The committee is multi-disciplinary and includes ethics, economics, clinical oncology, and decision-makers • Discussion of uncertainty and its impact on decision-making is articulated • Decision is based on the evidence package and associated uncertainty

#### Managed access

Managed access agreements allow patients to access new technologies while collecting additional data for RWE; for life-cycle HTA, the goal of managed access is to address key uncertainties and better inform reimbursement decisions. It is crucial to note that managed access agreements do not need to be promises of reimbursement but they do outline conditions under which reimbursement may be achieved. Managed access agreements can be informed by a combination of: decision-maker(s) conditions for allowing healthcare system access and industry sponsor conditions for funding health technology access; and the baseline value of initiating and continuing collection of RWD within the LHS using value of information analysis. While not extensively used in HTA, value of information analysis methods (e.g., expected value of sample information) can estimate the current expected value to society of collecting additional data from a sample of observations^31^. As such, the value of information analysis informs the time horizon of institutional support because it can quantify whether the value of collecting additional data is less (greater) than the cost of continuing to invest in research.

Key to supporting managed access and sustainable decision-making is set guidance regarding what constitutes comparative value. For an intervention to be considered cost-effective, decision-makers need to specify an endpoint, such as Net Monetary Benefit (NMB), which would need to be positive to demonstrate value for money. NMB represents the monetary value of an intervention conditional on both a particular willingness to pay for a health gain and the expected cost of technology implementation. This endpoint is calculated as the difference between incremental cost and the product of incremental effectiveness and payers’ willingness to pay for a gain in effectiveness, i.e., NMB = λ*ΔQALY − Δ*C*. Where Δ*C* is the cost difference between the treatment and comparator, termed incremental cost (*C*_t_ − *C*_c_), ΔQALY is incremental effectiveness (QALY_t_ − QALY_c_), and λ is payers’ willingness to pay for a QALY gain. QALYs are anchored on preference-based values between 0 (death) and 1 (perfect health). The willingness to pay threshold (λ) represents the opportunity cost of displacing QALYs from other interventions given budget constraints. For transparency, decision-makers will need to define λ. Decision makers will also deliberate on comparative clinical effectiveness. Determining the endpoint for clinical effectiveness will be disease-dependent, but patient-valued endpoints such as survival, health-related quality of life, or progression provide advantages over intermediate endpoints with uncertain patient utility (e.g., patient is matched to an experimental trial). When deliberating on clinical effectiveness, an endpoint with an established minimally important difference will facilitate deliberation. Understanding the impact of a defined clinical endpoint speaks to the importance of research with patients on their preferences and values.

#### Collecting and concatenating real world data

The specification of core data elements and how to achieve data collection is the second framework component, with RWD requirements informed by stakeholders and guided by the study design and analysis. Pollard et al. (2022) outline our consensus-based core data elements for precision oncology life-cycle assessment, emphasizing the importance of collecting data throughout the patient disease and care trajectory, including the time period prior to life-cycle HTA study initiation^32^. The core data elements necessary for life-cycle assessment are in Supplementary Table 1. Data spanning the pre-study time period enables RWE analyses that generate synthetic control cohorts. Synthetic control cohorts can include historical controls and controls from different jurisdictions, with data on the entire patient trajectory allowing for statistical analyses of patient outcomes that can adjust for time-varying confounding and other biases.

Our life-cycle HTA framework responds to situations with small benefiting patient populations and low event rates by considering access to external datasets and pooling of cross-jurisdictional data. Life-cycle HTA also needs to be responsive to administrative data shortcomings. Administrative databases were established to support routine patient tracking and financial planning. Their use in life-cycle HTA has the advantage of being often population-based and generalizable to the routine unselected population, unlike cohort or clinical trials databases. On the other hand, they may not fully capture the required data for analyses. Inconsistently collected data, missing variables, and incomplete data entry introduce additional challenges for comparative evaluations. Resultant effect estimates may be subject to both random and systematic error, unless life-cycle HTA considers methods for addressing and imputing missing data.

External data generated through cohort studies or clinical trials provides an opportunity for integration and linkage with health systems-generated RWD. Leveraging existing datasets (Fig. 2) will allow for initial analyses to be conducted based on current evidence and can facilitate components of the prospective data analysis, through informing intervention outcomes (e.g., variant identified and treatment given) or usual care outcomes (e.g., variant identified and treatment not given) based on individual-level data. As we will show next, careful thought has to be given to study design and analyses generating RWE from non-randomized cohorts.

#### Real world evidence generation

**Fig. 3:**
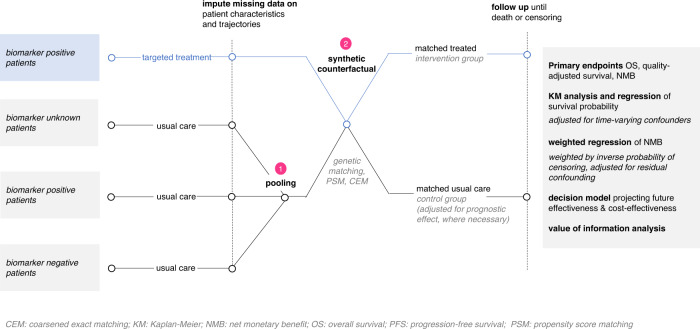
Example observational study design for precision oncology. The example design is a retrospective matched cohort study analyzing linked routinely and prospectively collected data from multiple sources. This study design addresses missing data using multiple imputation and integrates usual care patient data to inform a synthetic counterfactual for treated patients. Matching identifies this counterfactual, with matching method selection based on sample sizes, confounding sources, and maximization of covariate balance. Comparative outcomes are then established.

Life-cycle HTA requires an approach to study design adaptable to continuous data collection and iterative evaluation. Figure 3 presents an example non-RCT study design for evaluating effectiveness and cost-effectiveness of CGP-directed precision oncology targeting a rare biomarker. The study design is a retrospective cohort study analyzing linked routinely and prospectively collected data from multiple sources. The relevant study period spans the patient’s entire disease trajectory, from disease onset to date of intervention, to death or end of follow-up, with final outcomes analyses that allow for left- or right-censored data. With managed access, the primary cohort comprises consenting patients eligible to undergo CGP testing for a targeted therapy. The intervention group includes patients with a rare biomarker identified by CGP who receive targeted treatment. If the targeted therapy also requires the new reimbursement of a CGP technology, the intervention group may also include those who receive CGP but are biomarker negative. The comparison group are those with or without the rare biomarker who do not receive the targeted treatment. Given that patients with a rare biomarker who receive usual care are usually unobserved, quasi-experimental methods, pooling of available data from across cohorts or jurisdictions, and consideration of prognostic effects are needed to inform a synthetic counterfactual with adequate statistical power for effect detection.

Within-sample of the real-world evaluation, matching methods, such as propensity score matching, coarsened exact matching, or machine learning (e.g., genetic matching) can identify controls similar to the intervention group, who instead received usual care^28^. We caution that quasi-experimental approaches cannot adjust for all types of unmeasured confounders, emphasizing the importance of planning in advance to ensure that the LHS collects all necessary data elements to help avoid selection bias. Missing data, another source of bias, again speaks to the need of planning ahead when designing the LHS platform. Other potential biases for causal inference of CGP interventions are lead time bias or immortal time bias, in which treated patients must be outcome-free until their treatment date and thus have improved relative outcomes as well as time-dependent effects of subsequent lines of cancer therapy (systemic, radiation, or surgery)^33^. These can be minimally addressed through analytic methods and study design, built on an awareness of the clinical context of each case. Stakeholders should note that there is currently no optimal quasi-experimental method for adjusting for unmeasured confounders using observational study designs and a risk of bias will always be present. Externally, individual-level data can be leveraged to estimate and prognosticate the trajectories of those patients known to be biomarker positive or negative, but who did not receive the therapy. Further, clinical trial data from industry stakeholders can be leveraged to inform biomarker-positive patients who received the drug intervention.

Non-parametric and parametric methods for effectiveness and cost-effectiveness analyses on the entire cohort need to be agreed upon and applied periodically over time. Both Kaplan-Meier survival analysis and regression models can be considered to estimate incremental differences in endpoints, such as overall survival and quality-adjusted survival, within the time horizon of the available data^34^. Heterogeneity in clinical effectiveness across patient subgroups should be explored through stratified analysis or pooled analysis when sample sizes permit. For evaluating cost-effectiveness, regression-based methods based on NMB can be used. These methods have the advantages of being able to adjust for residual confounding and to characterize covariate impacts on marginal cost-effectiveness^35,36^. In the likely presence of censoring, net-benefit regression may be weighted by participants’ inverse probability of censoring weights to reduce estimation bias^37^. Additionally, decision modelling is a key tool that enables projection of future effectiveness and cost-effectiveness over a longer time horizon; it accounts for the patient trajectory beyond the observed intervention period. Conservative approaches for modelling should be used, including the assumption of no added health or survival benefit beyond the observed real-world trial period. Value of information analysis can identify which parameters are driving decision uncertainty and inform continued data collection and reimbursement decisions^31,38^.

#### Interpretation of the evidence and update decision

Decision-making is usually concerned with static estimates of efficacy, expected net present value of net benefit, and budget impact or feasibility, conditional on the current state of knowledge. Life-cycle HTA is a continuous decision-making process with the need for ongoing re-assessment informed by data emergence and horizon scanning. At pre-specified intervals, an interdisciplinary arms-length and independent prioritization committee should examine the updated evidence that is tailored for the deliberative processes. In Canada, prioritization committees exist both federally and within jurisdictions. Given life-cycle HTA is responsive to small sample sizes where high decision uncertainty is present, we suggest that an initial managed access recommendation is made at the national level, followed by coordination of data generation and prioritization at both the provincial and federal levels. The specifics around the managed access approach and the coordination of data sharing is a topic of ongoing debate and is a crucial area of additional research. Regarding data sharing between jurisdictions, we believe technology-enabled federated analysis is an important step for producing cross-jurisdictional evidence where the RWD does not have to leave the jurisdiction within which it was generated.

The decision to disinvest, to continue with evidence development, or to fund a technology (Fig. 2) should be based on previous knowledge and on the new evidence emerging from life-cycle evaluation. As discussed, the value of information analysis can inform whether to collect additional evidence. The value of collecting additional evidence will also be shaped by the committee’s tolerance for uncertainty of clinical effectiveness and the joint uncertainty in incremental costs and benefits.

Life-cycle HTA deliberations that are not supported by RCT evidence face the critical issue of potentially biased outcomes estimation. As a result, life-cycle HTA utilizing RWD absent of randomized study protocols should be subject to considerable scrutiny and oversight. We recommend that independent HTA outcomes units collaborate with decision-making committees to define the endpoints, study protocols, and supporting analyses needed for deliberation. Further, research infrastructure that allows for scrutinization of safety, effectiveness, and cost outcomes and that permits requests of additional analyses by experts will be an important component for ensuring trust in RWE. We recognize that HTA units will need to collaborate with for-profit entities, particularly when patient-level data from clinical trials to support RWE are key to addressing uncertainty and when financial resourcing may be required. Ideally, publicly and privately funded life-cycle HTA experts will work together to inform clinical trial protocols. This important collaboration is critical for enabling transparent life-cycle HTA analyses and subjecting the protocols and analytic approaches to scientific debate.

## Conclusion

In summary, the adoption of a life-cycle HTA framework alongside transparent and comprehensive decision-making, is needed to implement an LHS approach for CGP-informed precision oncology. This approach will permit a deeper understanding of the value of precision oncology, and allow a continued agile response to the constantly changing landscape of precision oncology innovations. We encourage further research into how healthcare systems can be equipped to have sufficient personnel and infrastructure to achieve the goal of LHS supporting life-cycle HTA.

## Supplementary information

Supplementary Table 1

## Data Availability

Data sharing not applicable to this article as no datasets were generated or analysed during the current study.

## Code availability

Code sharing not applicable to this article as no coding or algorithms were used during the current study.
