# Supplementary material for: A perspective on life-cycle health technology assessment and real-world evidence for precision oncology in Canada
Source: NPJ Precis Oncol. 2022 Oct 25;6:76. doi: 10.1038/s41698-022-00316-1 (PMC9596463; doi:10.1038/s41698-022-00316-1)
Supplement: Supplementary file 1 — Supplementary Table 1 [file 41698_2022_316_MOESM1_ESM.pdf]

Supplementary Table 1 – Checklist of core data elements required for life-cycle HTA in precision oncology

Category/ Data Element	Item No.	Recommended timeline for data collection	Included (Y/N)
**Demographic and socio-economic factors**			
Unique patient identifier	1	Baseline	
Date of birth or age	2		
Sex	3		
Location (e.g., region, postal code, local health authority)	4		
**Clinical characteristics**			
Tumour group	5	Baseline	
Tumour subgroup	6		
Histology (e.g., tumour grade)	7		
Date(s) of all primary cancer diagnoses established through pathology and/or imaging	8		
Site specific staging criteria (e.g. TNM)	9		
At least one performance status measure (e.g., Eastern Co-operative Oncology Group (ECOG) performance status)	10	At diagnosis, baseline, and ongoing at regular intervals	
Date(s) of cancer recurrence established through pathology and/or imaging	11		
Date(s) of cancer metastasis established through pathology and/or imaging	12		
**Genomic elements**			
Date(s) and type(s) of prior genetic testing received	13	Baseline (historical)	
All historical genetic test reports (including single gene and germline mutation tests)	14		
Date(s) of patient's tumor biopsy	15	At occurrence	
Date(s) patient's normal DNA comparator was collected (e.g. blood sample)	16		
Flag for whether biopsy site was metastatic	17		
Flag for whether biopsy site was radiated	18		
Pathology tumour content from biopsy	19		
Genomic tumor content from biopsy sample	20		
Flag sufficiency to undergo sequencing	21		
Date of bioinformatics report	22		
Aggregated sequencing information from bioinformatics report (e.g. tumour mutation burden, immune signature)	23		
Actionable findings (e.g. OncoKB, European Society for Medical Oncology (ESMO) scale, etc.)	24		

Category/ Data Element	Item No.	Recommended timeline for data collection	Included (Y/N)
Informative findings (e.g. feature or mutation that may not have prognostic or therapeutic relevance at the time of analysis)	25		
Relevant genes for which a germline variant was identified and corresponding pathogenicity	26		
Sequencing type (e.g. genome, transcriptome, exome, multi gene expression testing)	27		
Date(s) that a genetic diagnosis was established	28		
Cost of clinical consult	29		
Cost of sample acquisition & preparation (e.g. anesthesia, sample collection, pathology reagents)	30		
Cost of next generation sequencing	31		
Cost of bioinformatics analysis (including computation, analyst time)	32		
Cost of validation and confirmatory testing	33		
Cost of interpretation by committee	34		
Number of pre- and post- NGS genetic counselling appointments	35	At first occurrence, ongoing	
Cost of genetic counselling appointments	36		
**Cancer treatment – Systemic therapy**			
Number of lines of therapy received	37	Historical & ongoing	
Date(s) lines were received	38		
Treatment protocol(s)	39		
Drug name(s)	40		
Treatment intent (e.g. curative or palliative)	41		
Access indicator, if applicable (e.g. off-label, clinical trial, out of pocket)	42		
**Cancer treatment – Surgical**			
Date(s) of surgical treatment	43	Historical & ongoing	
Body site of surgical resection	44		
Treatment intent of surgery (e.g. curative or palliative)	45		
**Cancer treatment – Radiotherapy**			
Date(s) of radiotherapy treatment	46	Historical & ongoing	
Radiotherapy body site, dose, and fractionation	47		
Modality of radiotherapy (e.g. SABR, IMRT, VMAT, 3DCRT, Brachytherapy)	48		
Treatment intent of radiotherapy (e.g. curative or palliative)	49		
**Cancer Treatment - All types**			

Category/ Data Element	Item No.	Recommended timeline for data collection	Included (Y/N)
Indicator if treatment was provided pre- or post-sequencing	40	At first occurrence, ongoing	
Indicator if treatment was genomics informed	51		
Reason why genomics-informed treatment was not given, if applicable	52		
**Patient outcomes**			
At least one preference-based measure (e.g. EQ5D, Health Utility Index (HUI), EORTC QLQ C3015)	53	Baseline & ongoing at routine intervals	
Death date	54	At occurrence	
Disease-specific clinically relevant secondary endpoints, as applicable	55	At first occurrence & ongoing	
Date(s) of disease progression, established through [e.g. Response evaluation criteria in solid tumours (RECIST and iRECIST) criteria, clinician assessment]	56		
Clinician assessed best response on genomics-informed and usual care cancer treatment, (e.g.: Stable disease, Complete response, Partial response, or progression, Not evaluable)	57		
Costs of cascade genetic testing and intervention(s)	58		
**Resource utilization**			
Type and dates of hospitalizations pre- and post-NGS (admissions and discharges, including ER and ICU)	59	Historical & ongoing	
Costs of hospitalizations pre- and post-NGS	60		
Type and dates of physician visits pre- and post-NGS (e.g. General practitioner, Oncologist, Other specialist)	61		
Costs of physician visits pre- and post-NGS	62		
Type and dates of imaging (e.g. CT, MRI, PET, Ultrasound, X-ray)	63		
Costs of imaging pre- and post-NGS	64		
Type and dates of non-genomic lab tests	65		
Costs of non-genomic lab tests	66		
Type and date of non-cancer prescription drugs	67		
Costs of non-cancer prescription drugs	68		

The above table reports a checklist for all data elements required to support cost-effectiveness evaluations in precision oncology. This table was modified based on the following article with author permission: *Pollard S, Weymann D, et al. Defining a core data set for the economic evaluation of precision oncology. Value in Health. 2022. PMID: 35216902 DOI: 10.1016/j.jval.2022.01.005*
